# How I do it: Lung ultrasound

**DOI:** 10.1186/1476-7120-12-25

**Published:** 2014-07-04

**Authors:** Luna Gargani, Giovanni Volpicelli

**Affiliations:** 1 Institute of Clinical Physiology, National Research Council, Via Moruzzi, 1, 56124 Pisa, Italy; 2 Department of Emergency Medicine, San Luigi Gonzaga University Hospital, Orbassano, Torino, Italy

**Keywords:** Lung ultrasound, B-lines, Point-of-care ultrasound, Chest sonography

## Abstract

In the last 15 years, a new imaging application of sonography has emerged in the clinical arena: lung ultrasound (LUS). From its traditional assessment of pleural effusions and masses, LUS has moved towards the revolutionary approach of imaging the pulmonary parenchyma, mainly as a point-of-care technique. Although limited by the presence of air, LUS has proved to be useful in the evaluation of many different acute and chronic conditions, from cardiogenic pulmonary edema to acute lung injury, from pneumothorax to pneumonia, from interstitial lung disease to pulmonary infarctions and contusions. It is especially valuable since it is a relatively easy-to-learn application of ultrasound, less technically demanding than other sonographic examinations. It is quick to perform, portable, repeatable, non-ionizing, independent from specific acoustic windows, and therefore suitable for a meaningful evaluation in many different settings, both inpatient and outpatient, in both acute and chronic conditions.

In the next few years, point-of-care LUS is likely to become increasingly important in many different clinical settings, from the emergency department to the intensive care unit, from cardiology to pulmonology and nephrology wards.

## What is lung ultrasound?

**Figure 1:**
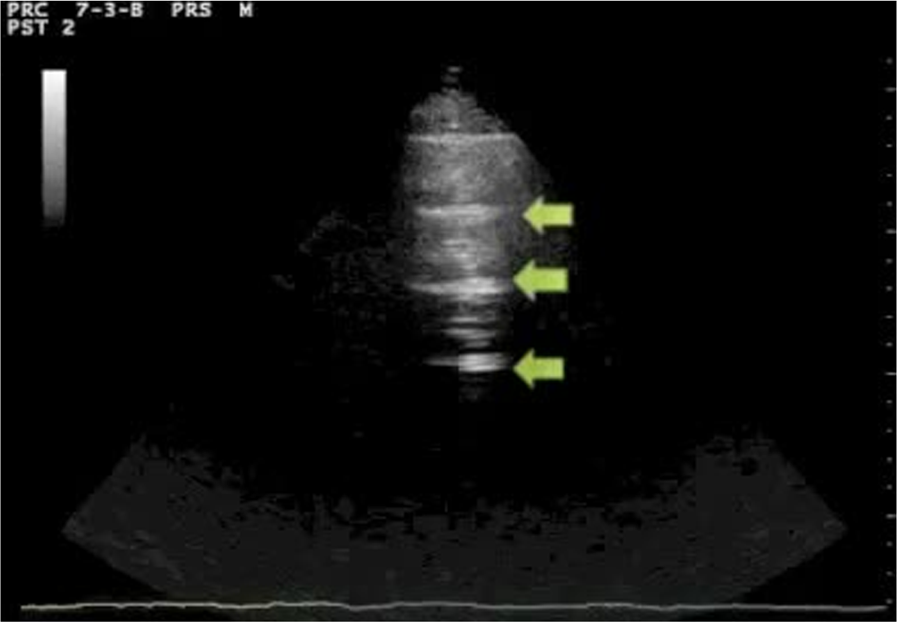
**Sonographic appearance of an aerated lung scan.** Arrows indicate A-lines. Above A-lines the pleural line is visible with its horizontal movement, the lung sliding.

**Figure 2:**
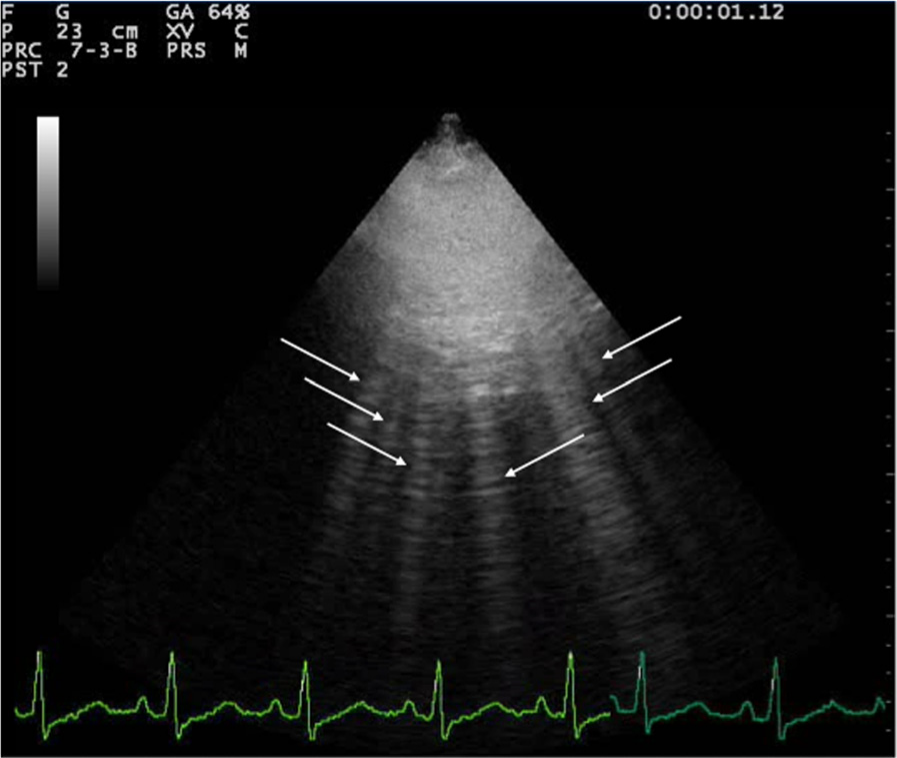
Sonographic appearance of multiple B-lines (indicated by the white arrows).

**Figure 3:**
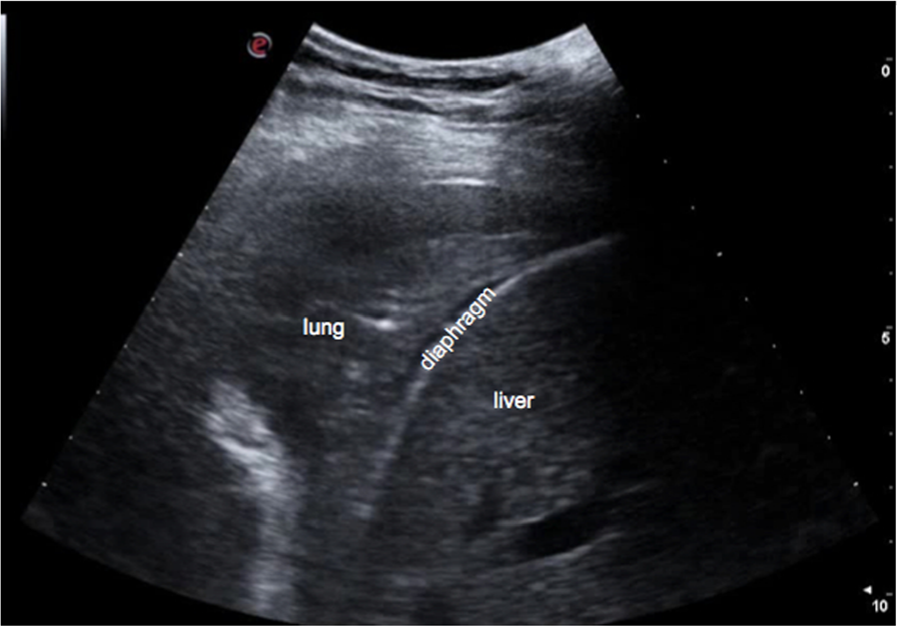
**Sonographic appearance of a consolidated lung.** The echo-texture of the lung becomes similar to the liver.

Assessment of the lung has always been considered off-limits for ultrasound, since it is standard textbook knowledge that «because ultrasound energy is rapidly dissipated by air, ultrasound imaging is not useful for the evaluation of the pulmonary parenchyma» [1]. The concept that ultrasound cannot be employed for evaluating the lung is linked to the presence of air, which determines a high acoustic mismatch with the surrounding tissues, causing a complete reflection of the ultrasound beam, preventing the creation of direct imaging of the pulmonary parenchyma [2]. In a normally aerated lung, the only detectable structure is the pleura, visualized as a hyperechoic horizontal line. It is debated whether this line represents an artefact due to a reflection phenomenon at the interface between alveolar air and the soft tissues of the thoracic wall, or it images the real pleura. The pleural line moves synchronously with respiration [3]: this dynamic horizontal movement is called *lung sliding*. In addition, there are some hyperechoic, horizontal lines arising at regular intervals from the pleural line: the A-lines. When combined with lung sliding, these reverberation artefacts represent a sign of normal or excessive content of air in the alveolar spaces (Figure 1, Additional file 1). When the air content decreases and lung density increases due to the presence in the lung of exudate, transudate, collagen, blood, etc. the acoustic mismatch between the lung and the surrounding tissues is lowered, and the ultrasound beam can be partly reflected at deeper zones and repeatedly. This phenomenon creates some vertical reverberation artefacts known as B-lines (Figure 2, Additional file 2). B-lines belong to the family of the comet-tail artifacts, well known in the setting of abdominal ultrasound [4]. B-lines have also been addressed as comet-tail artifacts or ultrasound lung comets before an expert agreement on nomenclature was obtained [3]. B-lines are defined as discrete laser-like vertical hyperechoic reverberation artifacts that arise from the pleural line, extend to the bottom of the screen without fading, and move synchronously with lung sliding [3]. Multiple B-lines are considered the sonographic sign of lung interstitial syndrome, and their number increases along with decreasing air content and increase in lung density [5,6]. When the air content further decreases, such as in lung consolidations, the acoustic window on the lung becomes completely open, and the lung may be directly visualized as a solid parenchyma, like the liver or the spleen (Figure 3). Consolidation of the lung may be the result of an infectious process, an infarction due to pulmonary embolism, a localization of cancer and metastasis, a compression or obstructive atelectasis, or a contusion in thoracic trauma. Additional sonographic signs may help determine the aetiology of the consolidation, such as the quality of the deep margins [7], the presence of air or fluid bronchogram [8], or the vascular pattern within the consolidation [9].

The acoustic limitations of ultrasound in the assessment of an air-rich organ such as the lung can paradoxically become a diagnostic advantage. In some conditions the presence of air between the chest wall and the lung parenchyma causes a decisive change of the dynamic characteristics of the sonographic artefact image of the lung described so far. In pneumothorax (PNX) lung sliding is always absent [10], since it can be observed if the lung and the parietal pleura are in direct apposition, but not when the physical acoustic enemy – the air – is between the two pleural layers. For similar reasons, no B-lines can be seen in the context of a PNX, since B-lines can be visualized only at an air-tissue acoustic interface, when the visceral pleura is opposing the parietal pleura. Another sign helps rule out PNX, the *lung pulse*, which refers to the subtle rhythmic movement of the lung upon the parietal pleura, synchronous with cardiac beats [11]. Like the respiratory movement, this cardiac movement of the lung cannot be detected by ultrasound if air is present between the visceral and parietal pleura. An easy step-by-step sonographic algorithm has been proposed to diagnose/exclude PNX by LUS [3,10].

In summary, LUS may be defined as a powerful diagnostic imaging technique for anomalies of the pleural space [12] and a reliable densitometer of the lung parenchyma [5]. This definition of LUS includes both its virtues, which should be included in clinical practice, as it is often time-, cost- and potentially life-saving; as well as its limitations, which should never be forgotten for a correct use of this technique.

## How I do it: the setting, the scanning technique, the probe

**Figure 4:**
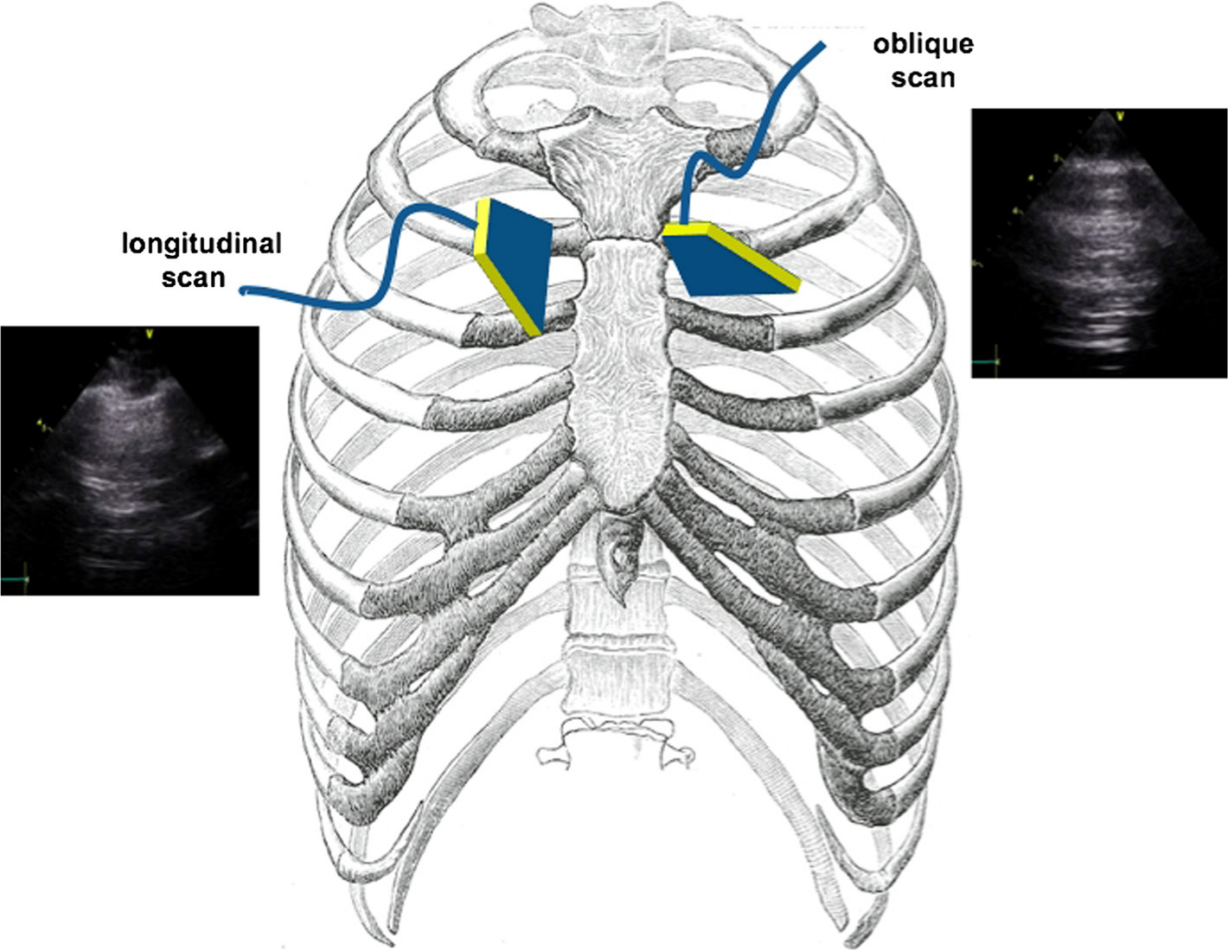
Longitudinal and oblique approach to lung ultrasound.

**Figure 5:**
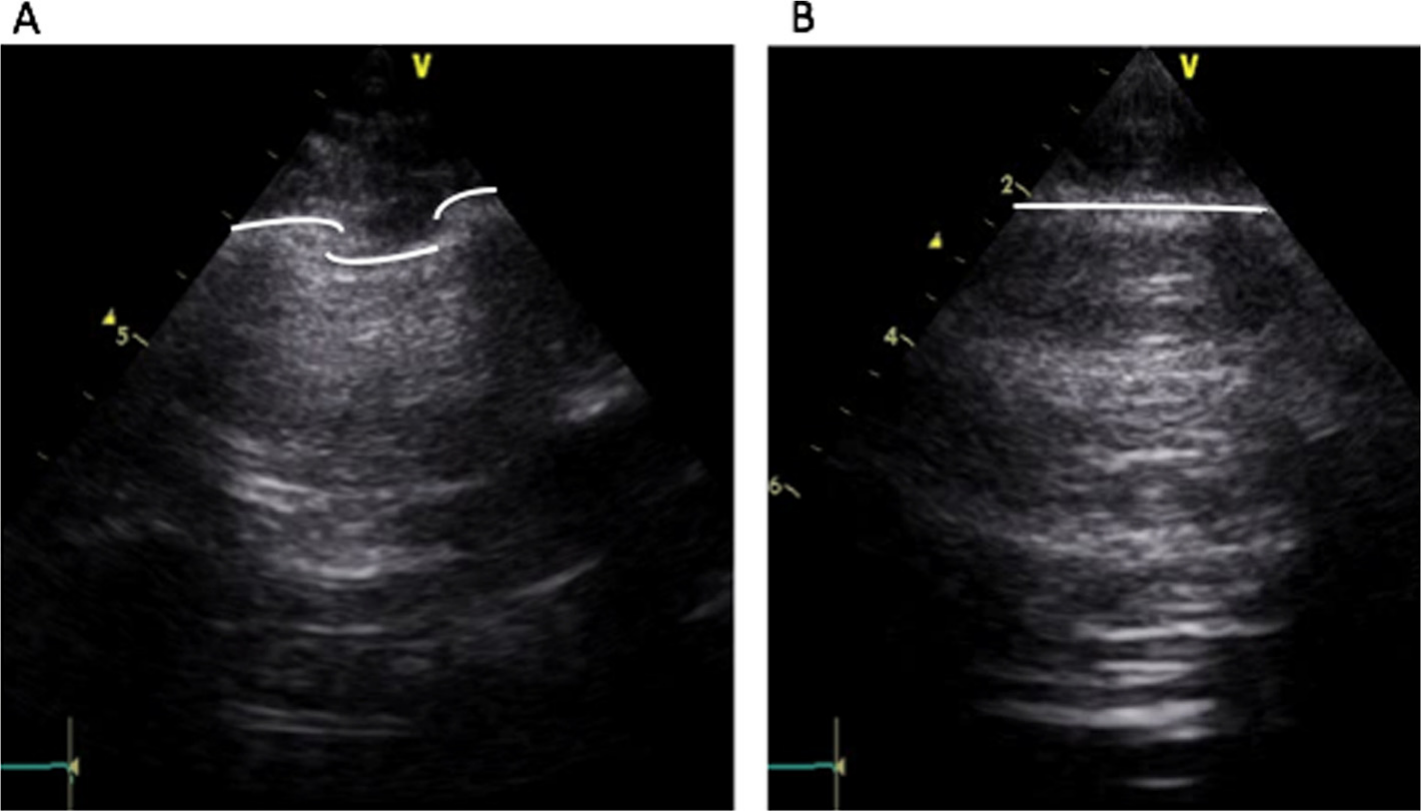
**Longitudinal and oblique lung scanning. A.** Longitudinal lung scanning: the upper rib, the pleural line and the lower rib draw an image that resembles a bat. **B.** Oblique lung scanning: the pleural line is not interrupted by the ribs, and appears as a horizontal line.

LUS can be performed on the whole chest, just laying the probe in the intercostal spaces, avoiding the ribs. The probe can be positioned both longitudinally, perpendicular to the ribs, and obliquely, along the intercostal spaces (Figure 4). The longitudinal approach allows visualization of the so-called “bat-sign” (Figure 5). In a longitudinal view the bat sign identifies the upper and lower ribs (the wings of the bat) and, a little deeper, the pleural line (the back of the bat). The oblique approach allows visualizing a larger part of the pleural line, which is not interrupted by the rib shadows (Figures 1 and 5).

The diagnostic approach based on LUS can vary according to different settings and clinical situations, following the main principles of what is today known as “point-of-care ultrasound”. Maximum effectiveness of the method is obtained through a clinically-driven and focused assessment. If properly driven and correctly interpreted, some sonographic signs become highly accurate for diagnosing specific pulmonary conditions. For example, in a stable patient with acute spontaneous pleuritic pain, ultrasound examination will start from the painful chest area [13], focusing on signs of focal pleural and parenchymal abnormality. If the pain is caused by a pulmonary condition with involvement of the parietal pleura, this will be easily detected by LUS. Indeed, LUS is a surface imaging technique, highly sensitive in detecting pleural abnormalities. The clinical suspicion and pre-test probability will guide the diagnostic process to rule in or rule out with high accuracy several conditions, such as PNX, pleuritis, pneumonia, lung peripheral infarction [14]. In this setting, a highly specific sign is the *lung point*, which represents the transition point between the typical sonographic pattern of PNX (absence of lung sliding and of B-lines) into the normal pattern of lung sliding, and depicts the physical limit of PNX as mapped on the chest wall [3]. However, the lung point can be employed to detect the extension of PNX, but not its volume. Up-to-now, LUS is not recognized as a method to differentiate between large and small PNX.

**Figure 6:**
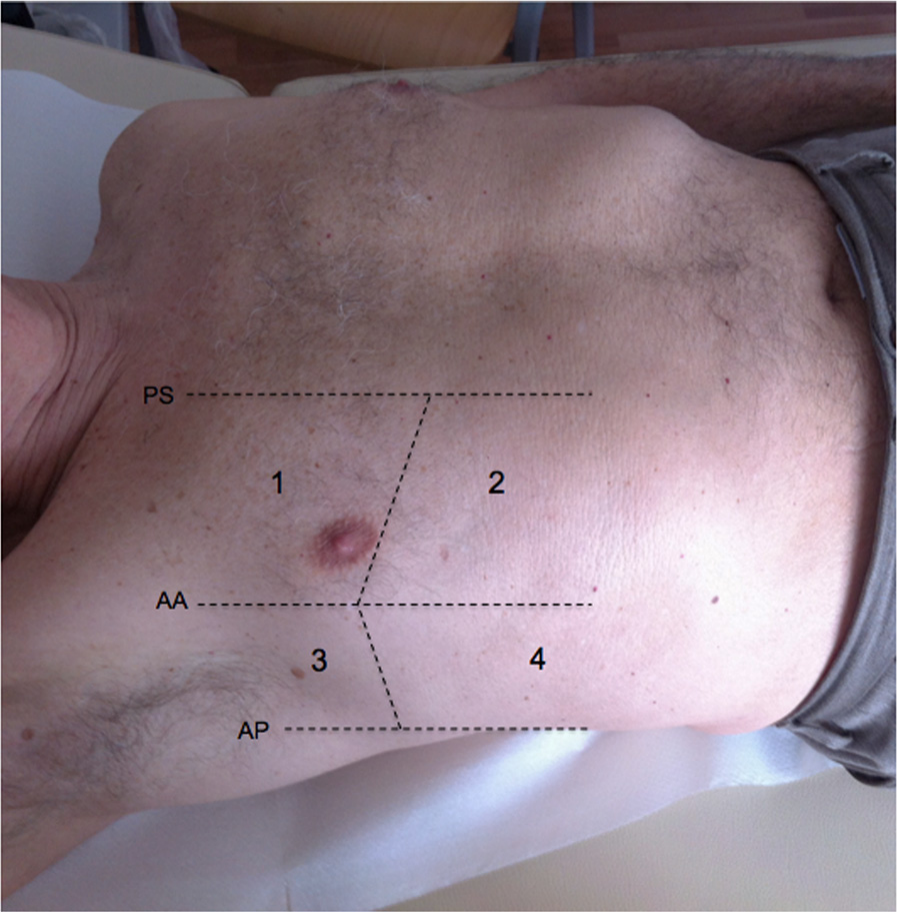
**Eight-zone scanning scheme of the antero-lateral chest (according to Volpicelli G et al. See ref. **[15]**).**

In a patient with acute dyspnea, if cardiogenic pulmonary edema is in the differential diagnosis, LUS will be used to examine the anterior and lateral chest to detect the diffuse signs of interstitial and alveolar edema, which usually respect three highly specific features: they are correlated with the severity of the respiratory failure, follow a regular and symmetric spatial distribution, and usually progress from the lateral and inferior (dependent zones) to the anterior upper chest areas. The scanning technique that should be employed in the emergency setting is the eight-zone examination, consisting of scanning four chest areas per side (Figure 6): areas 1 and 2 denote the upper anterior and lower anterior chest, whereas areas 3 and 4 denote the upper lateral and basal lateral chest, respectively [15]. In the critically ill patient with acute respiratory failure, a more rapid anterior two-region scan may be sufficient to rule out the interstitial syndrome due to cardiogenic acute pulmonary edema [16]. However, this focused anterior scanning, while still highly accurate in the critically ill, may not be sufficient in patients who are not severely dyspnoic [17], since presence of B-lines on the anterior chest usually denotes a more severe degree of pulmonary congestion in case of heart failure. Again, this is another example of adaptation of the LUS technique and signs to the specific setting and clinical condition.

If the main clinical suspicion is the possibility of a PNX, the LUS examination is started from the non-dependent zones for air collection, corresponding to the anterior-inferior chest in the supine patient. In this case, examining only one *hot zone* per side will help rule out PNX with high sensitivity both in the extreme emergencies and in stable patients. The hot zone examination will also be enough to confirm PNX in unstable/cardiac arrest patients [18]. Only in stable patients will examination be extended to the lateral chest to confirm PNX.

**Figure 7:**
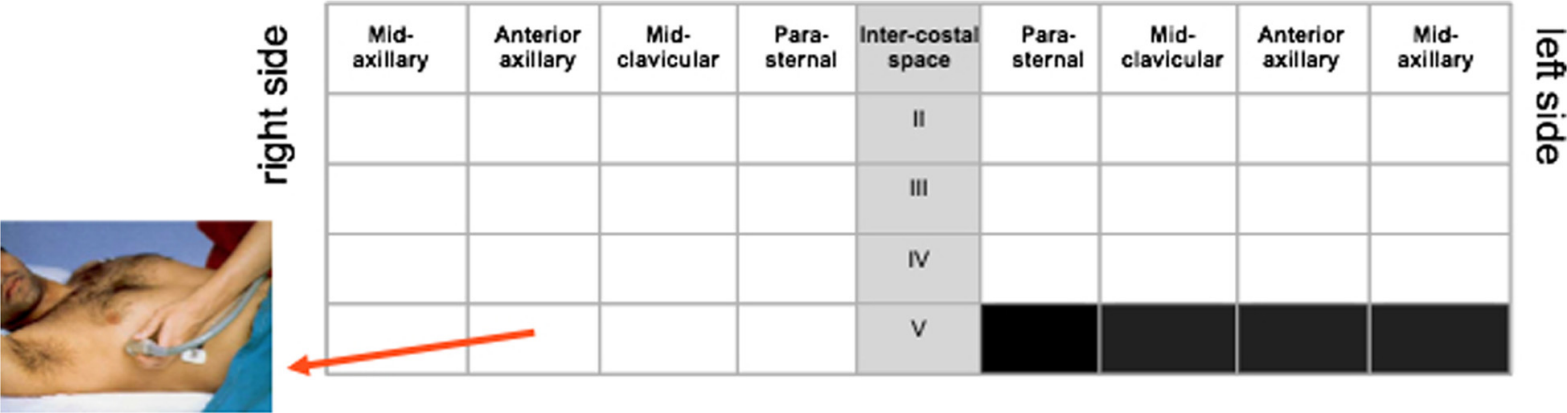
**Twenty-eight scanning site scheme of the antero-lateral chest (Modified from Jambrik et al. See ref. **[46]**).**

**Figure 8:**
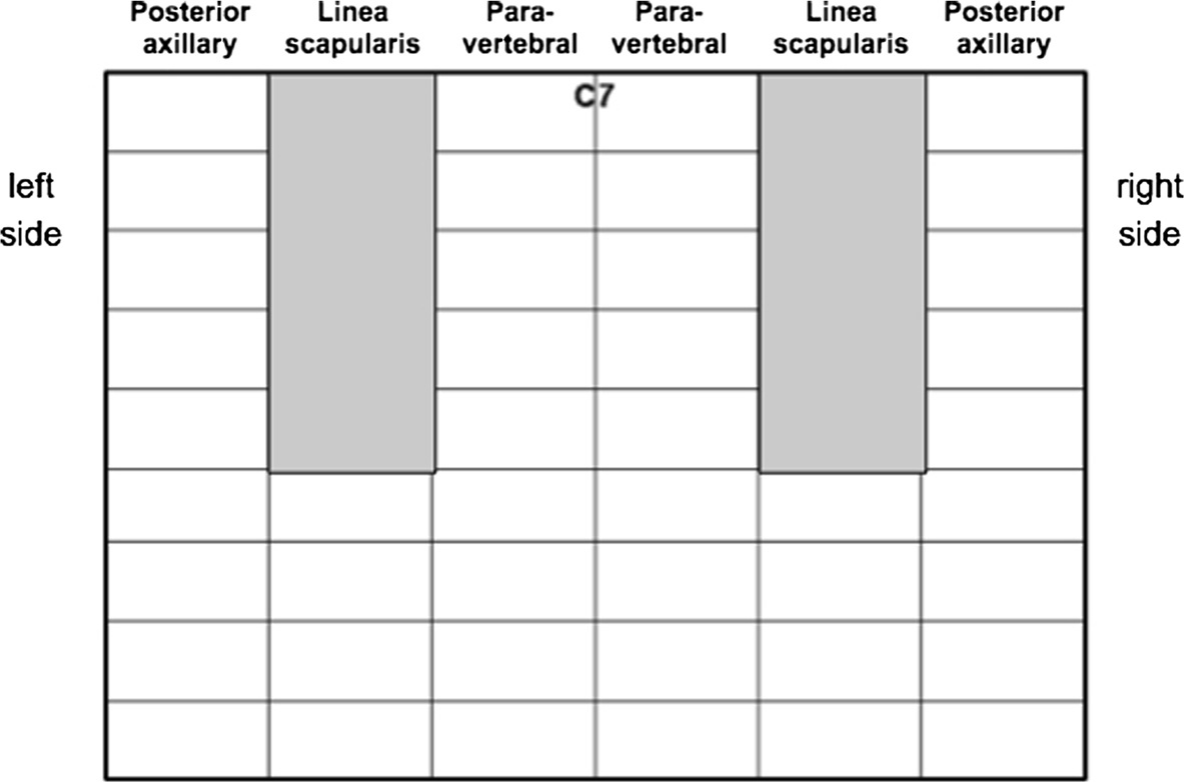
**Scanning scheme for the posterior chest (Modified form Gargani L et al. See ref. **[24]**).**

In the setting of chronic patients, with less time pressure and more borderline cases, the scanning technique should always be more comprehensive. It can include the anterior, lateral and dorsal chest. Different approaches have been proposed: a detailed scanning scheme has been used in many studies on patients with heart failure [19,20], on dialysis [21-23], and with pulmonary fibrosis [24,25], focused on the assessment of B-lines. These approaches allow accurate examination of the whole chest, which can be applied in several settings in chronic conditions. It is particularly useful for quantifying the extent of the LUS abnormalities, and for assessing intra-patient variations after therapeutic interventions, including dialysis [21,22]. Ultrasound scanning of the anterior and lateral chest is obtained on the right and left hemithorax, from the second to the fourth (on the right side to the fifth) intercostal spaces, and from the parasternal line to the axillary line (Figure 7). The posterior chest is scanned along the paravertebral line, linea scapularis and posterior axillary lines (Figure 8). The sum of the B-lines found on each scanning site yields a score denoting the extent of the pulmonary interstitial syndrome. Zero is defined as a complete absence of B-lines in the investigated area. When B-lines are a few in a scanning site, they can be easily counted. When they are more numerous, they tend to be confluent and it is less easy to clearly enumerate them. To obtain a semiquantification of the sign, you can consider the percentage of the scanning site occupied by B-lines (i.e., the percentage of white screen compared to black screen below the pleural line) and then divide it by ten (i.e., 30% corresponds to about 3 B-lines, 70% corresponds to about 7 B-lines, and so on) [26].

**Figure 9:**
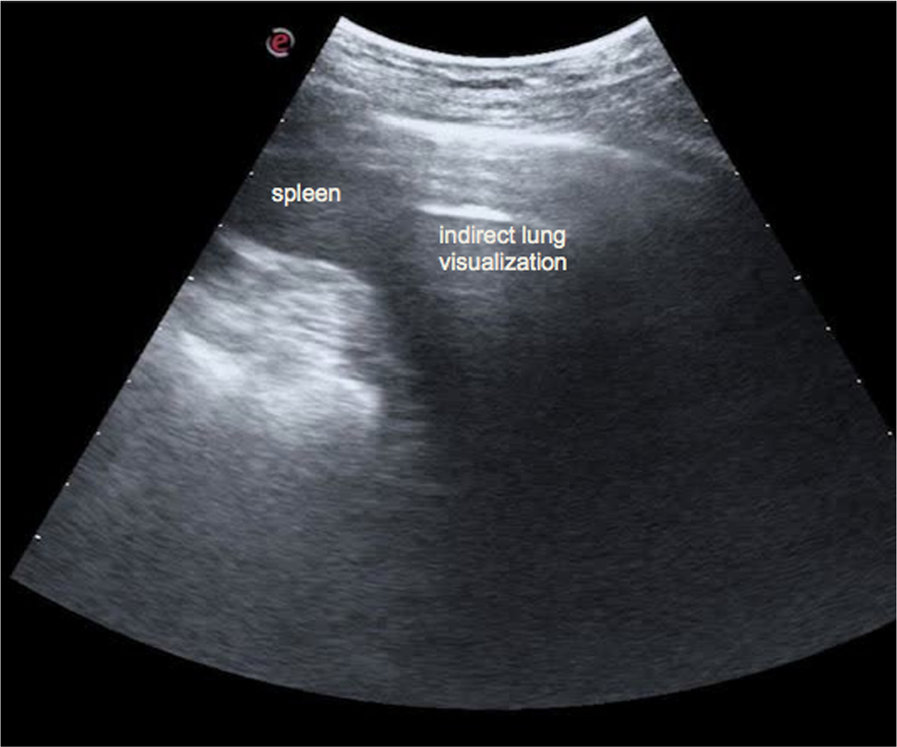
**Left costophrenic angle.** During inspiration the lung moves downward and the lung air prevents the visualization of part of the spleen.

**Figure 10:**
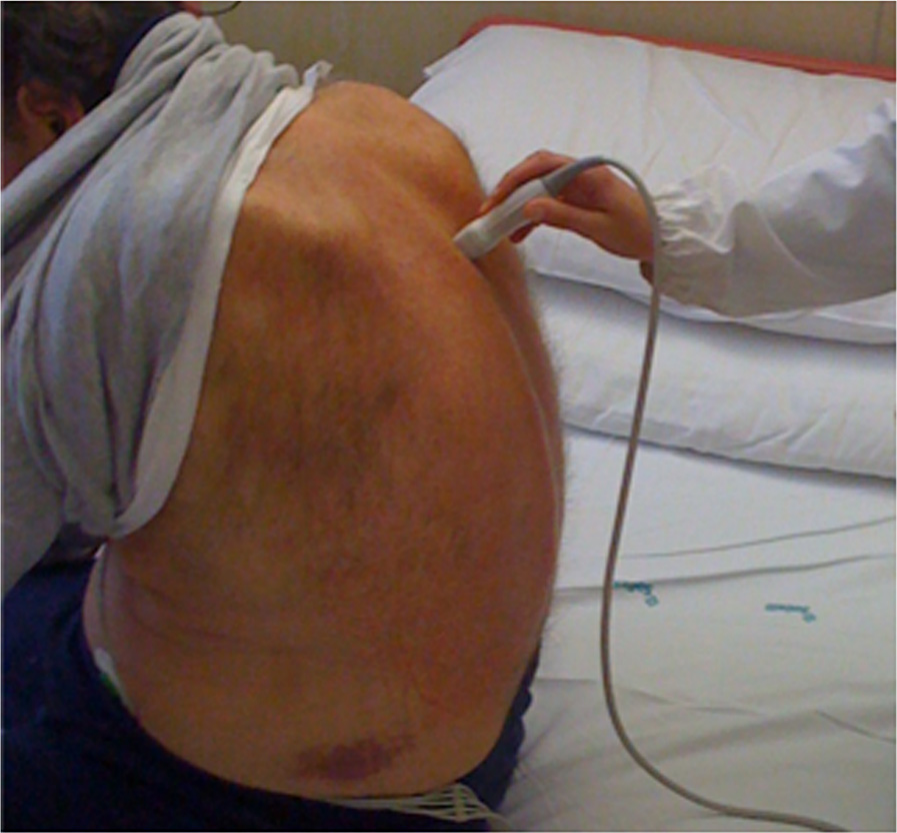
Position of the patient to scan the posterior chest.

In the case of limited time, even in a chronic setting the examination can be more focused and should be clinically driven. In patients with heart failure, it is important to scan the dependent zones, i.e., lung posterior basis if we are evaluating an out-patient, or along the posterior and mid-axillary lines if we are scanning an in-patient who has been lying down for many hours. In patients with interstitial lung disease such as pulmonary fibrosis, it is mandatory to scan the posterior chest, because the disease generally starts in this region. It is advisable to always scan the costo-phrenic angles on both sides, and to assess the “curtain sign”, that is the relative movement of the lung, which covers the subdiaphragmatic organ during inspiration (the liver on the right side, or the spleen on the left side) and the diaphragm (Figure 9 and Additional file 3). This is the region where free pleural effusion can be detected. Identification of pleural effusion is the most established application of LUS [1,3,27]. The effusion should first be sought in dependent zones, i.e., lateral and posterior chest. By LUS it is possible to differentiate pleural effusion from atelectasis, consolidations, masses, or an elevated hemidiaphragm, that sometimes may hardly be distinguished on a chest X-ray. LUS images pleural effusion as an anechoic or hypoechoic space between the two pleural layers, with the lung appearing either aerated or consolidated. LUS for the diagnosis of pleural effusion is especially valuable in critically ill patients, showing better sensitivity and reliability than bedside chest X-ray [28-30]. Ultrasound can detect the effusion, evaluate its volume, provide information on its nature, and indicate the appropriate area for thoracentesis. Moreover, LUS outperforms CT scan in the diagnosis of complex effusion due to its ability to distinguish septa and fibrin inside the collection [31].LUS can be performed in any position (supine, lateral decubitus, or prone), since lung abnormality distribution does not change so rapidly that significant information would be missed (apart from pleural effusion) just by changing the patient’s position. Moreover, in the case of pulmonary congestion, even if the position of B-lines changes, the overall distribution tends to remain the same, without clinically relevant differences. The supine position is perfect for scanning the anterior chest, whereas the lateral chest may be examined in the semi-supine position (on the left decubitus to scan the right axillary lines, and on the right decubitus to scan the left axillary lines). The ideal position for scanning the posterior chest is with the patient sitting on the bed, his/her back turned to the operator (Figure 10). Indeed, it is possible to also scan patients while they are standing, sitting or lying flat, without significant differences in results. The only real limitation is when LUS needs to be extended to the dorsal chest of a patient lying down who is intubated in the intensive care unit, or a patient who is unconscious and cannot be moved. In these situations, using small probes that may be better placed between the bed and the patient may allow the best result.

The LUS examination can be performed using any commercially available 2-D scanner. Different transducers have been used, such as phased array (cardiac), convex (abdominal), microconvex, and linear (vascular) probes. Higher frequencies and macro probes are useful for the evaluation of the pleural line and subpleural space, so should be preferred for assessing PNX. Phased-array probes can be successfully employed to detect pleural effusion, thanks to low frequency and consequent ability to provide a deeper view of the chest. However, these latter probes have limitations in detecting PNX and when a detailed examination of the sub-pleural space is needed. The convex and microconvex probes are the most universally used, all-purpose probes for LUS, thanks to their intermediate frequency values, which allow a reasonable visualization of the pleural line and subpleural space, without losing the overview of the chest. B-lines can be detected by all these different probes, but again low frequency probes are probably the best for this application. Although the number of B-lines may be slightly different when using different probes in a specific chest site, the overall clinical picture does not change by changing the transducer [32]. The possibility of easily assessing B-lines with any kind of transducer is one of the advantages of this technique, so no one should give up on scanning a patient just because the “ideal” probe is not available.

Portable machines and pocket-sized devices have also been proposed for assessing B-lines, as well as pleural effusion [33-35]. There is no need for a second harmonic or Doppler imaging mode, so even older ultrasound machines can be employed. Visualization of lung consolidations is possible with all probes as well. In the case of a small consolidation, a phased-array transducer may offer less detail, whereas a linear transducer would magnify it. In the case of large consolidations, a linear probe may be unsuitable for detecting the consolidations’ borders precisely, whereas convex and microconvex, and even the phased-array transducers, would be more appropriate. The depth should be tailored to the patient: very thick ribcages, large muscles and obese patients need greater depths, even to visualize the pleural line. Very thin patients and children may require less depth. Depth should also be adjusted to the target of our examination: if we are looking for PNX, the depth should be lower, in order to better visualize the pleural line and assess the presence or absence of the sliding sign. If we are looking for a free pleural effusion, the depth should be greater, for a better overview of the costo-phrenic angles. Normally the focus should be positioned at the pleural line level, but it should be moved deeper when our main target is less superficial.

## How I do it: interpretation

Interpretation of LUS images is usually not very challenging. We must keep in mind that LUS is more affected by lack of specificity than lack of sensitivity. A LUS pattern showing absent lung sliding or multiple B-lines or a lung consolidation may be not enough to establish a specific diagnosis, since it can be linked to different pathologic conditions [36]. Indeed, this limitation in specificity is a common feature of several diagnostic tools that we routinely interpret in daily clinical practice, from physical examination to EKG, from chest X-ray to more sophisticated instrumental findings. The power of these tools resides in the interpretation of signs when combined with each other at bedside, together with a consideration of the overall clinical picture. When all patient characteristics are taken into account, including history, symptoms, physical examination, setting, comorbidity, medications, etc., specificity can increase significantly. For example, in a patient with systemic sclerosis and without any known left heart conditions, presence of multiple B-lines is more probably related to pulmonary fibrosis than to extravascular lung water. On the other hand, presence of multiple diffuse bilateral B-lines in a patient with reduced cardiac function is more likely to be related to extravascular lung water than to fibrosis [26].

**Figure 11:**
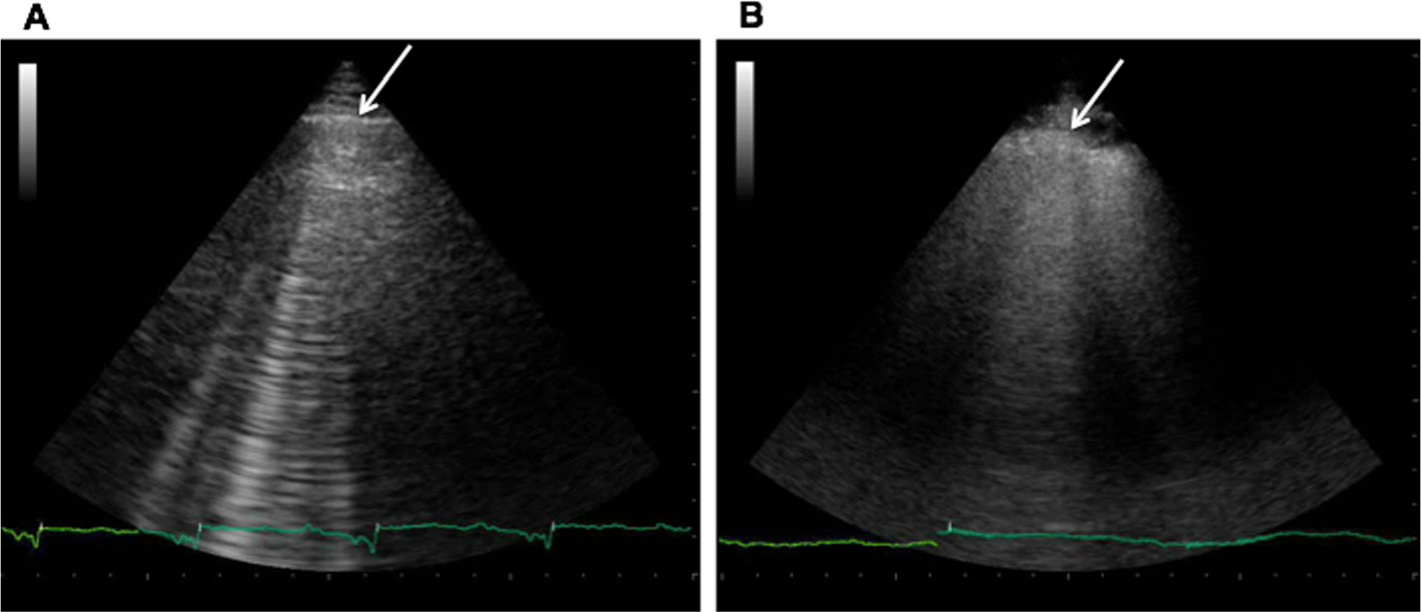
**Multiple B-lines in cardiogenic pulmonary edema and lung fibrosis. A.** Multiple B-lines in a patient with cardiogenic pulmonary edema: the arrow indicates a normal pleural line. **B.** Multiple B-lines in a patient with pulmonary fibrosis: the arrow indicates the abnormal pleural line, which looks irregular.

The clinical condition of the patient is probably the most important feature that helps interpret LUS findings and influence patient management. For instance, in an unstable patient with signs and symptoms of hemodynamic shock or cardiac arrest, the absence of any movement of the pleural line, either respiratory (lung sliding) or cardiac (lung pulse), coupled with absence of B-lines raises such a high suspicion of PNX that may lead to placement of a chest tube, even without the need for a more extensive ultrasound examination or for other diagnostic techniques [10]. On the other hand such a simplified protocol is not advisable in a stable patient, where there is time to extend the examination looking for adjunctive signs that enhance the specificity of the ultrasound diagnostic process. Distribution of B-lines and pleural line characteristics are also crucial to increasing the specificity of LUS. B-lines due to cardiogenic pulmonary edema are usually bilateral, start appearing in the dependent zones and usually diffusing or recovering symmetrically. B-lines due to pulmonary fibrosis generally start at the posterior lung basis, and are often associated with irregularity of the pleural line and subpleural small consolidations (Figure 11). In contrast to pulmonary edema due to congestion or overhydration, acute lung injury/ARDS shows a dishomogeneous and irregular pattern, featuring many subpleural consolidations, highly fragmented pleural line and intense hyperlucent multiple B-lines alternating with spared areas [37]. This irregular distribution of B-lines contrasts with that observed in cardiogenic pulmonary edema, where B-lines are usually detected in more homogenous distribution, which is gravity-related, and it is quite rare visualizing subpleural consolidations. The presence of a lung consolidation with blurred margins in a patient with fever will raise high suspicion of pneumonia, whereas a triangular-shaped consolidation with absence of any color-Doppler signal, in a patient with chest pain and clinical risk factors for thrombo-embolic disease, will raise the suspicion of a peripheral pulmonary infarction. Dynamic response to therapy can also be useful for increasing the accuracy of LUS. In the case of multiple bilateral B-lines that resolve in days during ordinary treatment [38,39], or even in a few hours by an acute diuretic load [26,40], the cardiogenic or volume overload origin of B-lines is strongly suggested. Similarly, in end-stage renal disease patients, B-lines decreasing or even disappearing after either a hemodialysis [21,22] or peritoneal dialysis session [41,42], indicate pulmonary congestion due to overload.

Additional information deriving from a bedside focused ultrasound evaluation of other organs may also be helpful. This approach has been recently described in patients with undifferentiated hypotension, where the integrated point-of-care multiorgan ultrasonography of the heart, inferior vena cava, lungs and abdomen significantly agreed with a final clinical diagnosis obtained by retrospective chart review [43]. A multiorgan ultrasound approach including lung, heart and peripheral veins recently showed a better performance also for the diagnosis of pulmonary embolism than LUS alone [44,45].

A controversial issue is quantification of B-lines. In critically ill patients the assessment can be qualitative, since the ultrasound finding of acute conditions is usually well defined and clear. For instance, in a critically ill patient with acute respiratory failure, if the underlying condition is cardiogenic pulmonary edema, the sonographic appearance of the lungs will be striking, with multiple diffuse bilateral B-lines to convey a picture of “sonographic white lung”. In these patients, B-lines can also be found in the least dependent zones, i.e. the anterior chest. On the contrary, finding a limited number of B-lines (even if bilateral) in a very symptomatic respiratory failure patient should lead to excluding the diagnosis of a cardiogenic origin of the actual condition. In non-critical patients, a more careful assessment and quantification of B-lines may be useful, especially for the follow-up. As highlighted above, a semi-quantification of B-lines has been proposed [46] and subsequently used in many papers from different research groups [19-25,35,47-50]. For clinical purposes, the final number of B-lines can be categorized ranging from mild to severe degrees, similar to what is done for most echocardiographic parameters. This counting approach can be imprecise when considering single scanning sites, but nevertheless provides a reliable overall LUS picture, allowing more accurate monitoring of patients, both in acute conditions - i.e., rapid changes after diuretic therapy or dialysis [21-23] – but also in stable outpatients [20]. Moreover, this approach has shown good intraobserver and interobserver variability, consistently < 10% [22,46,51].

## How I do it: pediatric patients

Lung ultrasound can be very useful in neonates and children. The advantage in this population is related to the small size of the chest, which allows an optimal, although still indirect, visualization of the lungs. All LUS signs and patterns described in the adult are alike in neonates and children, in both normal and pathological conditions [52]. A number of studies have described the usefulness of LUS in the pediatric population, from transient tachypnea of the newborn [53] to respiratory distress syndrome [54], from bronchiolitis [55] to post-cardiac surgery lung complications [56] and anesthesia-induced atelectasis [57]. In the pediatric patients LUS is especially valuable in detecting pneumonia, with a sensitivity even higher than that of chest X-ray [58-60]. Given the small size of a child’s chest, a linear probe allows the best visualization of the lungs in most cases, irrespective of the depth of the main target of the examination. Considering their higher radio-sensitivity [61], children may especially benefit from a non-ionizing technique such as LUS, above all in chronic disease or during intensive care unit stay, where the cumulative radiation dose can reach high levels [62,63].

## Limitations

LUS limitations are essentially patient-dependent. Obese patients may be more difficult to examine due to the thickness of their ribcage and soft tissues. The presence of subcutaneous emphysema or large thoracic dressings alters or precludes the propagation of the ultrasound beams to the subpleural lung parenchyma.

It should be emphasized that LUS does not rule out pulmonary abnormalities that do not reach the pleura. This physical limitation is especially important when ruling out consolidations, since some consolidations, especially in the case of tumors, can be medially located and surrounded by aerated lung, which will prevent their visualization by sonography. The pulmonary interstitial syndrome from different etiologies sometimes may spare (although rarely) the subpleural space. A focal interstitial syndrome can sometimes be the “peripheral alarm” of a more medial pathological condition, for example in the case of peri-lesional interstitial edema, due to either inflammation or impaired lymphatic drainage.

## Conclusions

While application of ultrasound for the detection of pleural effusions and masses is well established, the sonographic assessment of the lung parenchyma is relatively new. We can perform LUS for evaluating both lung parenchyma and pleural space quite easily, after a relatively brief learning curve that is significantly shorter than for other sonographic techniques, although it still requires proper training focused on the understanding of the ultrasound pulmonary semiotics and the correct clinical interpretation of the LUS patterns. LUS is very suitable for a clinically driven, point-of-care assessment that should be tailored to the clinical suspicion and the setting. In the next few years this technique is likely to become the standard of care in several acute and chronic conditions.

## Consent

Written informed consent was obtained from the patients for the publication of this report and any accompanying images (Figure 6 and 10).

## Competing interests

The authors declare that they have no competing interests.

## Authors’ contributions

LG contributed to conception and design, and wrote the paper. GV; contributed to conception and design, and critically edited and revised the paper. Both authors read and approved the final manuscript.

## Supplementary Material

Additional file 1 **Sonographic appearance of an aerated lung scan.** Arrows indicated A-lines. Above A-lines the pleural line is visible with its horizontal movement, the lung sliding. Click here for file

Additional file 2 Sonographic appearance of multiple B-lines (indicated by the white arrows). Click here for file

Additional file 3 **Left costophrenic angle.** During inspiration the lung moves downward and the lung air prevents the visualization of part of the spleen. Click here for file
